# Siamese Networks-Based People Tracking Using Template Update for 360-Degree Videos Using EAC Format †

**DOI:** 10.3390/s21051682

**Published:** 2021-03-01

**Authors:** Kuan-Chen Tai, Chih-Wei Tang

**Affiliations:** 1 InnoFusion Technology, 3F-10, No. 5, Taiyuan 1st street, Zhubei, Hsinchu 30288, Taiwan; kuanchen0@gmail.com; 2 Communication Engineering Department, National Central University, Taoyuan 320317, Taiwan

**Keywords:** people tracking, 360-degree videos, equi-angular cubemap (EAC), siamese networks, timing detector of template update, machine learning, dimension reduction

## Abstract

Rich information is provided by 360-degree videos. However, non-uniform geometric deformation caused by sphere-to-plane projection significantly decreases tracking accuracy of existing trackers, and the huge amount of data makes it difficult to achieve real-time tracking. Thus, this paper proposes a Siamese networks-based people tracker using template update for 360-degree equi-angular cubemap (EAC) format videos. Face stitching overcomes the problem of content discontinuity of the EAC format and avoids raising new geometric deformation in stitched images. Fully convolutional Siamese networks enable tracking at high speed. Mostly important, to be robust against combination of non-uniform geometric deformation of the EAC format and partial occlusions caused by zero padding in stitched images, this paper proposes a novel Bayes classifier-based timing detector of template update by referring to the linear discriminant feature and statistics of a score map generated by Siamese networks. Experimental results show that the proposed scheme significantly improves tracking accuracy of the fully convolutional Siamese networks SiamFC on the EAC format with operation beyond the frame acquisition rate. Moreover, the proposed score map-based timing detector of template update outperforms state-of-the-art score map-based timing detectors.

## 1. Introduction

Visual tracking aims at locating and covering the target with an accurate bounding box. Among various kinds of videos, 360-degree videos feature panoramic information. Thus, they provide applications of computer vision rich environmental information and provide users an immersive viewing experience. Examples of applications of visual tracking on 360-degree videos are virtual reality (VR), augmented reality (AR), autonomous vehicles, unmanned autonomous vehicles (UAVs), health care, and medical image analysis. Although many visual trackers have been proposed, sphere-to-plane projection of 360-degree videos fails most in existing trackers since these trackers do not consider the characteristics of 360-degree videos.

The following subsections provide overviews of visual trackers on 360-degree videos (Section 1.1), Siamese networks-based visual trackers (Section 1.2), and template update for Siamese networks-based visual trackers (Section 1.3). Section 1.4. provides the motivation and contributions of the proposed scheme. All described trackers in Section 1 are summarized in Table 1.

### 1.1. Visual Tracking on 360-Degree Videos

**Figure 1:**
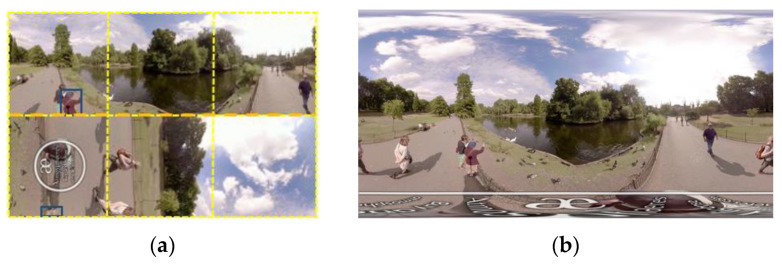
The 1330th frame of video #3 (London Park Ducks and Swans) [7]. (**a**) The equi-angular cubemap (EAC) format is a variant of the cubemap projection (CMP) format. The EAC format has six faces (yellow dashed line). The target is in the blue bounding box. The dashed orange line indicates boundary discontinuity between faces. Sphere-to-plane projection projects the target onto distant regions on an EAC image. Non-uniform geometric distortions occur in each face. (**b**) The ERP format has more severe non-uniform geometric distortions than the EAC format.

360-degree cameras have started to be commercialized in recent years. Although 360-degree videos-based novel applications are expected to become widespread in the near future, many challenges of 360-degree signal processing remain to be overcome [1]. In the latest video coding standard Versatile Video Coding (VVC) that was finalized in July 2020, several novel sphere-to-plane projection formats (e.g., equiangular cubemap (EAC) [2]) of 360-degree videos were considered [3]. These formats enable higher coding gain and higher tracking accuracy since they have less non-uniform geometric distortions. Few trackers have been designed for 360-degree videos using these formats since these formats are novel. Liu et al. proposed a convolutional neural network (CNN)-based multiple pedestrian tracker for 360-degree equirectangular projection (ERP) format videos [4]. The boundary handler extends the left/right boundary of a cropped panoramic image of ERP format. Bounding boxes extracted by a CNN-based object detector and are incorporated into an extended DeepSORT tracker to estimate the target state [5], where DeepSORT is in the framework of a Kalman filter. The cubemap projection (CMP) format and its variants have the problem of boundary discontinuity and deformation (Figure 1a). To solve the problem of boundary discontinuity and deformation of the CMP of road panorama captured by vehicle-mounted cameras, the pre-processing phase of the mean-shift-based tracker of rigid objects (e.g., car, traffic sign, or building) extends faces using the epipolar geometry that indicates the moving direction of the scene in [6]. To preserve edge continuity, one quarter (triangle) of the top image and one quarter (triangle) of the bottom image are stitched to the left/front/right/back image with pixel expansion.

### 1.2. Siamese Networks Based Visual Trackers

A target can be represented by handcrafted features or deep features. Handcrafted features are extracted using feature detection schemes with predefined algorithms and fixed kernels. Evaluation of precision-recall curves of several local handcrafted feature detection and description algorithms have been provided in [8]. On the other hand, by minimizing a loss function, a deep network framework that consists of linear layers (e.g., a convolution layer) and non-linear layers (e.g., an activation function) learns from a large scale of training dataset in the training phase. In the test phase, deep features are extracted using the trained network. Deep learning-based trackers benefit from learning with a huge dataset. The first deep learning tracker DLT adopted the stacked denoising autoencoder with off-line training and on-line fine-tuning [9]. Deep learning-based trackers can be categorized into two groups. The first group is based on tracking-by-detection. This group incorporates deep features that are taken as detection into a tracking framework. The second group follows tracking-by-matching, where one sub-group off-line trains the Siamese networks for similarity learning or regression. Siamese networks were proposed by Jane Bromley et al. in 1993 [10]. In the tracking phase, similarity between deep features of the target template and the candidate proposal in the current frame are computed to predict the bounding box of the target. Thus, trackers of the second group work well even the target sample has not been learned off-line. Moreover, it benefits from the small number of convolutional layers and thus it achieves real-time tracking. The classic and pioneer trackers of the second group are GOTURN and SiamFC [11,12]. Fully connected layers-based similarity computation of GOTURN (generic object tracking using regression networks) predicts coordinates of anchors of the bounding box [11]. SiamFC adopts the fully convolution network (FCN) where similarity is computed efficiently using cross correlation [12]. Such strategy enables the similarity to be computed in a translation manner. The output is a score map defined on a finite grid that indicates the likelihood of the target measurement. In SiamRPN [13], the Siamese subnetwork for feature extraction is followed by the region proposal subnetwork that includes regression and classification branches [13]. In the tracking phase, the input of the template branch is the template from the first frame. Then one-shot detection in the other frames is based on the category information from the template branch. Deep analyses of Li et al. found the lack of strict translation invariance of Siamese trackers [14]. Thus SiamRPN++ was proposed where the effective sampling strategy breaks the restriction. SiamRPN++ successfully enables training of the Siamese tracker with ResNet as a backbone network to obtain performance improvement [14]. Considering most Siamese trackers suffer from drifting problems due to learning global representation of the entire object, Liang et al. proposed LSSiam. Local semantic features are learned with additional classification branch off-line and then the classification branch is removed in the tracking phase [15]. Considering that semantic features learned for classification and appearance features learned for similarity complement each other, SA-Siam is composed of a semantic branch and a similarity branch [16]. Both branches are in the framework of Siamese network. The discriminative power of the semantic branch is improved by being weighted by an attention mechanism. The overall heat map (i.e., score map) of SA-Siam is generated using the weighted average of heat maps of two branches. In adaDCF [17], Fisher linear discriminant analysis (FDA) helps the tracker enhance discrimination between the positive (i.e., foreground) and negative (i.e., background) samples. A convolutional FDA layer is incorporated into the network framework with on-line fine tuning.

### 1.3. Template Update for Siamese Networks Based Visual Trackers

Although improvement of Siamese networks-based framework and loss functions helps learn visual representation off-line, template update plays a key role in the success of on-line tracking. SiamFC lacks online adaptability since it neither adopts on-line fine tuning nor template update [12]. Thus, it is not robust against geometric deformation and partial occlusions over time. A template is the exemplar that represents the target model (e.g., appearance model). In the tracking phase, template update replaces the original template with a new one to improve tracking accuracy. However, for a target with significant change of visual features over time, tracking with the target template extracted from the first frame suffers from drifting problems. Motivated by this, several template update schemes have been designed for Siamese networks-based trackers. For example, Valmadre et al. proposed a correlation filter-based deep tracker CFNet for end-to-end training [18]. The baseline Siamese networks-based tracker was modified with a correlation filter block to construct a discriminative template for robustness against translation, where a new template is generated for each new frame [18]. In Yang et al. [19], attentional Long Short-Term Memory (LSTM) control memory controls memory reading and writing. At each time instant, a residual template retrieved from the memory is combined with the initial template extracted from the first frame to build the new template. After Siamese networks-based target prediction, features of the cropped new target template corresponding to the predicted bounding box are written into memory for model update. Although the aforementioned template update schemes significantly improve tracking accuracy of SiamFC, an aggressive template updating strategy that does not introspect tracking accuracy easily leads to extra computation cost and drift problems. Moreover, the focus of these schemes is how to generate a new template instead of when to update template. Detection of the right timing of template update should be more efficient and effective. To deal with the problem that the annotation is available only in the first frame in the on-line tracking phase, clues extracted from tracking results can reflect tracking accuracy to some extent. Only a few schemes have proposed Siamese networks-based timing detector of template update. For example, Xu et al. use the highest value of a score map to determine the timing of update for UAV-based visual tracking [20]. If the value is small, conditions of deformation and partial occlusions will be further differentiated by the template update module, comprised of a contour detection network and a target detection network. If outputs of two networks are consistent, the template will be updated since deformation may occur. Otherwise, the template will be not updated to avoid the new template containing partial occlusions. For a Siamese networks-based score map, Wang et al. proposed the average peak to correlation energy (APCE) that denotes the ratio of the difference between the maximum and minimum value to the variation [21]. A higher APCE indicates the higher confidence of prediction being the target. When both the maximum value of the score map and APCE are greater than their weighted historical averages, the template is updated. In Liang et al. [15], proposed to detect the update timing by taking account into both the update interval and APCE-based confidence score [21].

### 1.4. Motivation and Contributions of The Proposed Scheme

Generally, a 360-degree image is stored as an ERP image after image acquisition and image stitching. However, it can be noticed that videos occupy 80% of internet traffic. Equiangular cubemap (EAC) format is one of variants of CMP format and it has less non-uniform geometric deformation than ERP (Figure 1) [2]. Thus, it enables more efficient video compression than ERP and higher tracking accuracy [22]. Each EAC image has six faces. All angles in the sphere domain have equal pixel counts in EAC format. Problems with tracking in EAC format are stated as follows. First, sphere-to-plane projection may make the target in the real world be projected onto distant regions on an EAC image. Accordingly, content discontinuity of EAC fails most state-of-the-art trackers. Second, to provide immersive viewing experiences, a 360-degree video has a huge amount of data. It is difficult to achieve real-time tracking. Third, non-uniform geometric deformation of EAC format makes the target appearance vary with frame index. Tracking accuracy will decrease if the template is not updated. Few visual trackers have been proposed for 360-degree EAC format videos. Since most handcrafted feature descriptors are not robust against non-uniform geometric deformation in the EAC format, Tai et al. proposed face stitching for the EAC format and a preliminary work of a timing detector of template update for the EAC format by referring to statistics of the score map [23], generated by SiamFC [12]. A total of 1600 local windows of score maps of eight 360-degree videos are analyzed. Analyses of observations indicate that a local window with high mean and low standard deviation implies the higher overlap ratio between the predicted bounding box and the ground truth of the target. However, thresholds of mean and standard deviation of a score map are selected for individual test videos heuristically in Tai et al. [23]. Thus, this paper proposes a Siamese networks-based people tracker using machine learning-based template update for 360-degree EAC format videos. Major contributions of the proposed scheme include: (1) regardless of the content discontinuity, the proposed tracker with face stitching can track a target that moves between any adjacent faces of EAC format. (2) The proposed machine learning-based timing detector of template update is built using a binary Bayes classifier, a statistically optimal classifier [24]. Statistical features and the linear discriminant feature of a score map, generated by the fully convolutional Siamese networks, are extracted and referred to. Statistical features include the mean and standard deviation. The linear discriminant feature is extracted using a dimension reduction Fisher linear discriminant (FLD) algorithm for statistical optimization [25]. The timing detector helps the tracker evaluate tracking accuracy without ground truth. Thus, the tracker can determine whether the template should be updated or not. (3) The proposed tracker operates at 52.9 fps, beyond the frame acquisition rate.

The remainder of this paper is organized as follows. Section 2 introduces the ERP plane-to-sphere projection and sphere-to-EAC plane projection. It also provides overviews of the classic fully-convolutional Siamese networks-based tracker, SiamFC [12]. Section 3 proposes the Siamese networks-based people tracker using timing detector of template update for EAC format. Section 4 provides experimental results and analyses. Finally, Section 5 concludes this paper.

## 2. Overview of Projection Format Conversion and Fully-Convolutional Siamese Networks Based Tracker

Before visual tracking on 360-degree EAC format videos, an ERP image is converted into the sphere domain and re-projected onto the planar representation of the EAC format. Thus, this section first states format conversion from ERP to EAC (Section 2.1). Next, the fully-convolutional Siamese networks-based tracker SiamFC is introduced (Section 2.2).

### 2.1. Conversion from Equirectangular Projection (ERP) to Equi-Angular Cubemap (EAC)

**Figure 2:**
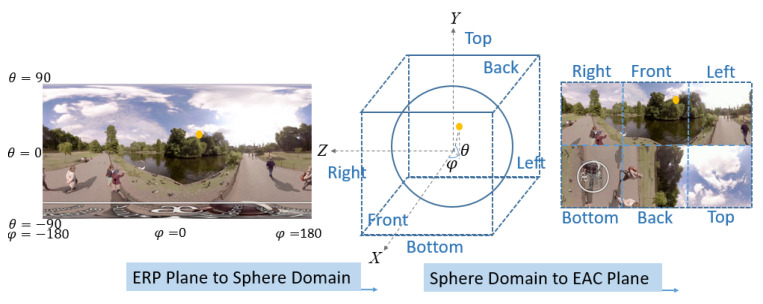
An example of conversion from the equirectangular projection (ERP) format to the EAC format for the 1330th frame of Video #3 (London Park Ducks and Swans) [7]. The orange dot denotes the location of a sample pixel.

Generally, a 360-degree image is stored as an ERP image after image acquisition and image stitching. To convert an ERP image into an EAC image, two phases are proceeded: ERP plane-to-sphere projection and sphere-to-EAC plane projection [2]. An example of conversion from the ERP format to the EAC format for the 1330th frame of Video #3 (London Park Ducks and Swans) is shown in Figure 2. In the first phase, a sampling point position (*i*, *j*) in an ERP image is converted to the position (*u*, *v*) in the 2-D projection plane by

(1) u=(i+0.5)/W,0≤i<W

(2) v=(j+0.5)/H,0≤j<H

where *W* and *H* are the width and height of an ERP image, respectively. Next, the longitude and latitude (φ,θ) in the unit sphere are calculated by

(3) φ=(u−0.5)×2π

(4) θ=(0.5−v)×π

(5) X=cos(θ)cos(φ)

(6) Y=sin(θ)

(7) Z=−cos(θ)sin(φ)

In the second phase, given the point position (*X*, *Y*, *Z*) in the sphere domain, the position $\left(u^{\prime },v^{\prime }\right)$ on the 2-D projection plane and the face index of an EAC image are computed [2]. Thus, the 2-D position $\left(\tilde{u},\tilde{v}\right)$ on an EAC image is calculated by

(8) $$\tilde{u}=(4.0/\pi )\times \tan^{-1}(u^{\prime })$$

(9) $$\tilde{v}=(4.0/\pi )\times \tan^{-1}(v^{\prime })$$

### 2.2. Fully-Convolutional Siamese Networks Based Tracker

The classic fully-convolutional Siamese networks-based tracker, SiamFC, was proposed by L. Bertinetto et al. [12]. The advantage of the Siamese networks is that they learn the similarity function of any pair of image patches where the convolution phase includes only several convolution layers. Thus, it is advantageous that it can track a target that has not been seen in the off-line training phase at high speed. SiamFC adopts neither on-line fine-tuning nor template update. Instead of fully connected layers-based regression, the cross-correlation-based similarity between feature maps of the template and those of the sub-image of the search image is computed efficiently since the operation is equivalent to use the inner product. The input of SiamFC includes the exemplar image *z* (i.e., the target template) and a candidate image *x*. With the aid of the VID dataset of the 2015 edition of the ImageNet Large Scale Visual Recognition Challenge (ILSVRC) dataset [26], the network learns the cross correlation-based similarity function f(z,x) that is defined as [12]

(10) f(z,x)=g(z)∗(x)+b

where g(⋅) is a convolutional embedding function, and b is a location independent real value. Implementation of g(⋅) in [7] follows the convolution architecture that consists of five convolution layers, two max pooling layers, and four ReLU (Rectified Linear Unit) layers of AlexNet [27]. Padding is avoided to keep the property of translation invariance of convolution. In the training phase, each convolution layer is followed by batch normalization to avoid the problem of an exploding/vanishing gradient. The output of the network is a score map defined on a finite grid $D\subset Z^{2}$.

The training phase aims at minimizing the mean of the logistic loss of training samples by updating parameters θ of the network:

(11) $$\arg\underset{\theta }{min}\underset{\left(z,x,a\right)}{E}L\left(a,f\left(z,x;\theta \right)\right)$$

where the logistic loss of a score map is defined as

(12) $$L\left(a,c\right)=\frac{1}{\left|D\right|}\underset{r\in D}{\sum }log(1+e^{-a\left[r\right]c\left[r\right]})$$

r is a 2-D position in the score map. a[r]∈{−1,+1} is the ground truth label of the position *r*, determined by the distance between the ground truth of the target position and the center of the candidate image. c[r] is the score corresponding to the exemplar image and the candidate image centered at the *r*th position within the search image. The stochastic gradient decent is adopted for optimization of Equation (11). In the tracking phase, the 2-D position with the highest score is taken as the predicted center of the target in the current frame.

## 3. The Proposed Tracking Scheme for EAC Format of 360-Degree Videos

At the off-line training phase, score maps are generated by applying Siamese networks-based tracker SiamFC to the dataset that consists of single-view videos [28]. Then the training dataset of the proposed timing detector is constructed using feature vectors of 100×100 sub-images, cropped from the image patch centering at the location with the highest score on a score map. Each training sample is labeled according to the overlap ratio between the predicted bounding box and the ground truth. After training the Fisher Linear Discriminant (FLD)-based projection vector for dimensionality reduction [25,29], each sub-image of a score map is represented by a feature vector including statistics of the sub-image and the FLD-based feature (Section 2.2). Then a binary Bayes filter-based timing detector is trained. Both the Fisher linear discriminant (FLD) and Bayes filter are supervised learning-based methods.

**Figure 3:**
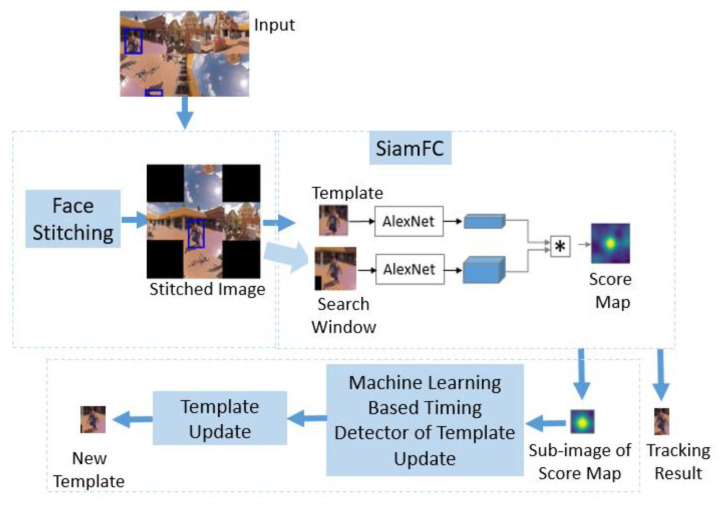
Block diagram of the proposed tracking scheme (tracking phase). The input is the 249th frame of Doi Suthep [7].

In the tracking phase of the proposed scheme (Figure 3), the target is manually detected with a tight bounding box drawn around the target in the first frame. Then the image patch with the context of the target is taken as the exemplar image (i.e., template). To be robust against content discontinuity between some adjacent faces, face stitching is applied before tracking (Section 3.1). SiamFC (Section 2.2) predicts the bounding box of the target in stitched images instead of original EAC images (Section 3.2). By referring to the predicted bounding box in the previous frame, a 100×100 sub-image of the current score map is cropped. Instead of an aggressive update strategy, the proposed machine learning-based timing detector of template update selects the right timing of the update (Section 3.3).

### 3.1. Face Stitching of EAC Format

Tai et al. proposed a face stitching scheme that successfully helps existing trackers to keep tracking on EAC images in [23]. Due to the sphere-to-EAC plane projection, the target in the sphere domain may be projected onto regions that do not belong to the same connected component in an EAC image. An example is shown in Figure 3, where the target (blue bounding box) in the sphere domain (real world) is projected onto disjoint regions in an EAC image (the 249th frame of Doi Sutheup [7]). Moreover, these disconnected components may be distant from each other. Accordingly, EAC images of 360-degree videos make most existing high-accuracy trackers fail. However, face stitching helps a tracker keep tracking across faces where borders are discontinuous. Moreover, the scheme has low computation complexity and avoids raising new geometric deformation in stitched images. The scheme is stated as follows.

**Figure 4:**
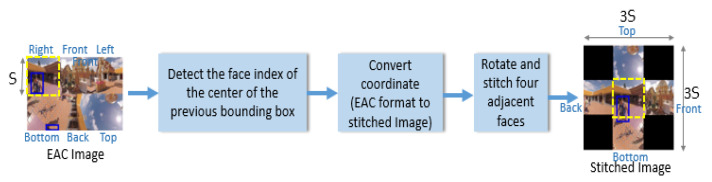
Block diagram of face stitching. The yellow face is taken as the central face. (EAC image: The 249th frame of Doi Suthep [7]).

**Figure 5:**
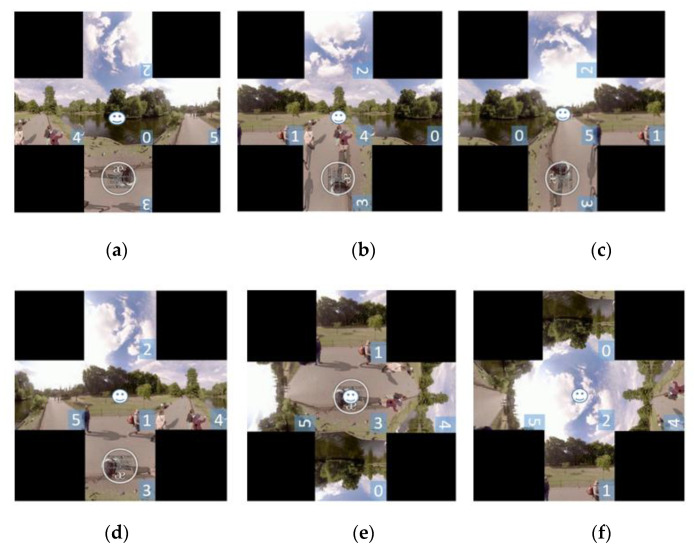
Stitched images that center at individual faces of the 1360th frame of London Park Ducks and Swans [7]. The face containing the smiling face indicates the target location. The orientation of the index number corresponds to the north–south axis in the sphere domain. The indices of central faces in the stitched images are (**a**) 0, (**b**) 4, (**c**) 5, (**d**) 1, (**e**) 3, and (**f**) 2, respectively.

An EAC image with six faces is indexed by {0,1,2,3,4,5}, where 0 denotes front, 1 denotes back, 2 denotes top, 3 denotes bottom, 4 denotes right, and 5 denotes left. In an EAC image acquired at frame rate either 30 fps or 60 fps [2], people usually cannot move from the current face to the opposite face of a cube in two consecutive frames. Motivated by this, the face stitching strategy only stitches four semantic neighboring faces in the sphere domain with the face indexed by N, where the center of the predicted bounding box of the target is located. Figure 4 shows the block diagram of face stitching. Let S denote the side length of a face. For the current frame, the central region (i.e., the central face) of a stitched square image with side length 3S is the face N that contains the center of the predicted bounding box in the previous frame. The central face indexed by 1, 2, or 3 is rotated upwards in stitched images. The four neighboring faces of the central face in the stitched image are the neighboring regions of the central face in the sphere domain. Regions containing four corners of the stitched image are filled with black pixels instead of extrapolated pixels from the existing faces. This is to avoid raising new geometric deformation in such regions. Figure 5 shows examples of stitched images that center at individual faces of the 1360th frame of London Park Ducks and Swans [7]. The face containing the smiling face indicates the target location. The orientation of the index number corresponds to the north–south axis in the sphere domain.

**Figure 6:**
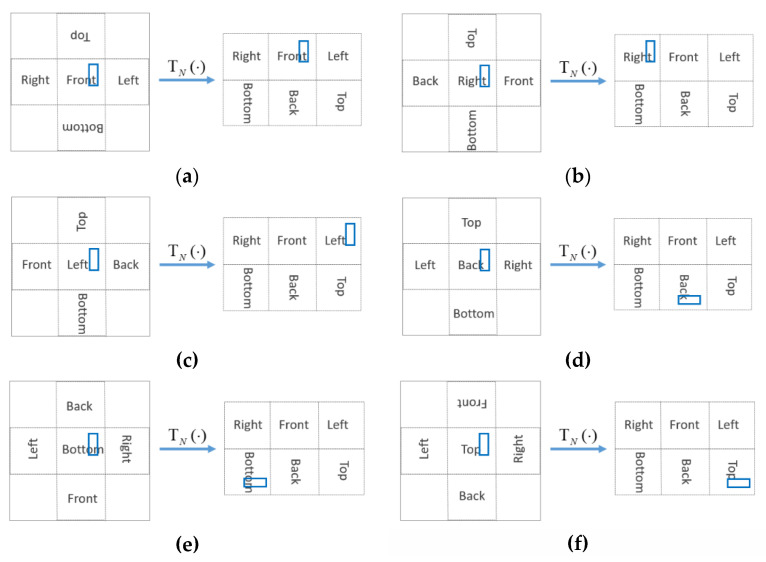
Conversion of the coordinate of the predicted bounding box of the target (blue) in different faces in the stitched image back to the coordinate in the EAC image by the *T_N_* (·). (**a**) Front. (**b**) Right. (**c**) Left. (**d**) Bac€ (**e**) Bottom. (**f**) Top.

At the tracking phase, the exemplar image (i.e., the target) and a search window (both in the stitched image domain) are input into the Siamese networks-based tracker. The coordinate of the predicted bounding box of the target in the stitched image is converted back to the coordinate in the EAC image by the function $T_{N}\left(\cdot \right)$, where the EAC image is packed in 3 × 2 format. That is,

(13) $$x_{N,e}=T_{N}\left(x_{s}\right)$$

where $x_{s}={\left[{\tilde{u}}_{s},{\tilde{v}}_{s}\right]}^{t}$ is the 2-D coordinate in the stitched image and $x_{N,e}={\left[{\tilde{u}}_{N,e},{\tilde{v}}_{N,e}\right]}^{t}$ is the 2D coordinate in the *N*th face of an EAC image. Figure 6 shows conversion for the target located in different faces. In some cases, the center of the predicted bounding box is not located in the central face of the stitched image. It indicates that the target just moved across different faces.

### 3.2. Feature Extraction of Score Maps Using Machine Learning Based Dimensionality Reduction

The adequate timing of template update is when the predicted bounding box of the target has high tracking accuracy. Thus, the problem of how to decide the update timing turns into the problem of how a tracker identifies the tracking result is good without knowledge of ground truth. Although Tai et al. found that mean and standard deviation of a sub-image of the score map of Siamese networks [23], SiamFC [12], were related to tracking accuracy, the mean and standard deviation-based update timing was decided for individual test videos manually and heuristically in [23]. Thus, this subsection first provides analyses of conditional probability distributions of the mean, standard deviation, and linear discriminant feature of score maps given the tracking result with high/non-high tracking accuracy. Next, this subsection proposes a statistics-based feature vector that consists of a mean, standard deviation, and dimensionality reduction-based feature of score maps for a machine learning-based timing detector of template update in Section 3.3.

**Figure 7:**
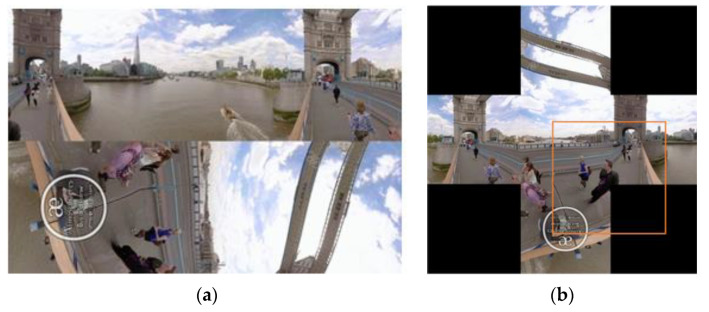
An example of a deformed and occluded target in the stitched image of EAC format of 360-degree videos. (**a**) An EAC image: the 274th frame of London on Tower Bridge [7]. (**b**) The stitched image with the center face containing the deformed target (the people in black) in (**a**). The search window of SiamFC is in orange. The padding pixels (black square) are similar to the occluding pixels on the target.

Face stitching solves the problem of content discontinuity between several adjacent faces of an EAC image. However, black padding pixels on four corners raised by face stitching lead to occlusions, similar to the one caused by the surroundings of the target (Figure 7b). In the meantime, non-uniform geometric deformation on boundaries of each face of EAC format still exists in a stitched image. However, compared with the ERP format and most variants of the CMP format, the EAC format features less non-uniform geometric deformations. Thus, this paper adopts single-view videos instead of 360-degree EAC format videos as training data of dimensionality reduction and a binary Bayes classifier for decision of update timing. Since the focus of this paper is people tracking, 21 videos containing people were selected from the OTB100 dataset [28]. These videos include Blurbody, Crossing, Crowds, Dancer, Dancer2, Girl, Gym, Human2, Human3, Human5, Human6, Human7, Human9, Jogging.1, Jogging.2, Singer1, Skating2.2, Subway, Walking, Walking2, and Woman. Videos containing people but with low tracking accuracy of SiamFC were not selected. The reason is that they cannot provide sufficient positive samples (i.e., score maps with high tracking accuracy). For selected videos, the number of training samples was approximately 10,000 while all 11 attributes (illumination variation, scale variation, occlusion, deformation, motion blur, fast motion, in-plane rotation, out-of-plane rotation, out-of-view, background clutters, low resolutions) of the OTB dataset were included. The trained SiamFC tracker was applied to each selected video. The tracker generates a score map for each video frame. A 100×100 sub-image centering at the location with the highest score on the score map is cropped. Each sub-image is taken as a training sample. A training sample will have the positive label if the tracking result of the corresponding video frame has a high overlap ratio (Section 3.2). Otherwise, it will have the negative label. Accordingly, the training dataset that contains 10,407 training sample is generated. This paper adopts the overlap ratio-based tracking accuracy as follows.

**Figure 8:**
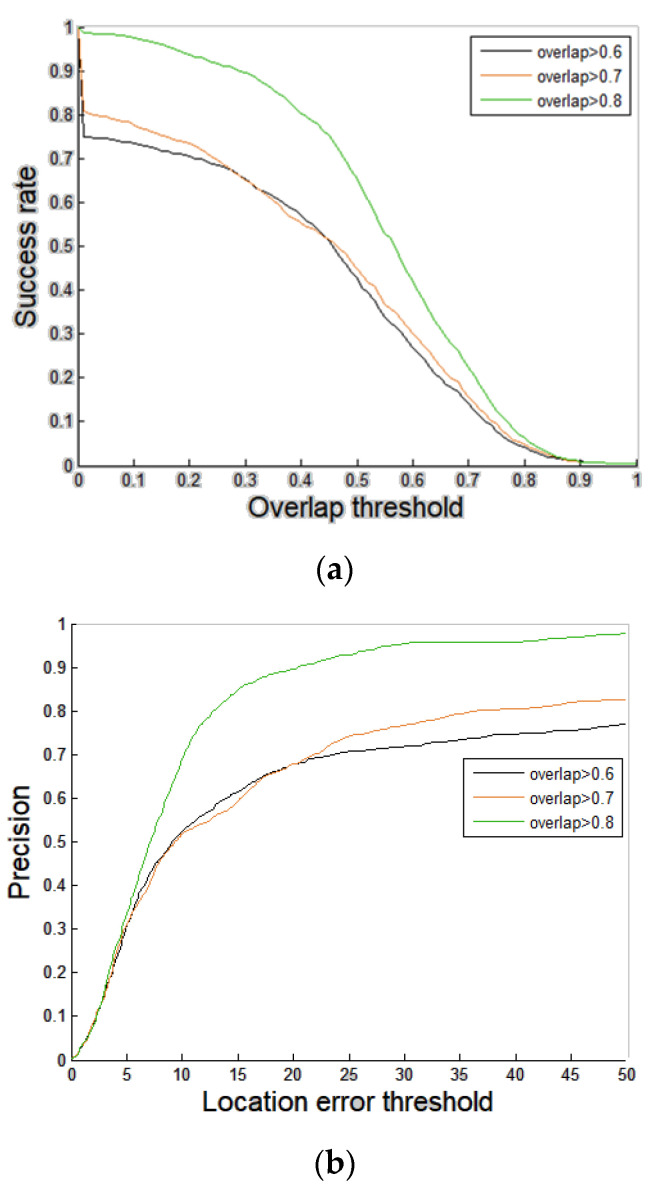
Comparisons among the proposed scheme with various settings of overlap ratio. (**a**) Success plots. (**b**) Precision plots.

First, the overlap ratio is defined by [28]

(14) $$O_{r}=\left|r_{p}\cap r_{g}\right|/\left|r_{p}\cup r_{g}\right|$$

where $O_{r}\in \left[0,1\right]$ is the overlap ratio. $r_{p}$ and $r_{g}$ are bounding boxes of the tracking result and ground truth, respectively. To improve tracking accuracy using template update, a new template that has a dissimilar appearance model with the original target template should be avoided. For example, an image patch that contains an occluded target or cluttered background is improper to use as a new template. It is suggested that the tracking result with $O_{r}$ that is greater than 0.6 is taken as high tracking accuracy in [13]. Although it will be the better update timing if the tracking result has higher accuracy (e.g., overlap ratio is 0.9), higher overlap ratio leads to infrequent update. Thus, comparisons of success plots and precision plots among the proposed tracking scheme with overlap ratios 0.6, 0.7, and 0.8 are shown in Figure 8. The success plot is generated using the overlap ratio between the predicted bounding box and the ground truth. If the overlap ratio is greater than a given threshold, the target will be taken as being tracked successfully in one frame. In the success plot, the success rate represents the ratios of successful frames at different thresholds that range between 0 and 1. The precision plot is generated using the Euclidean distance-based location error between the center of the predicted bounding box and that of the ground truth. The precision rate indicates the ratio of frames with location error that is less than a given threshold [28]. Tracking accuracy of the proposed scheme with overlap ratio 0.7 (orange curve) is similar to that of the proposed scheme with overlap ratio 0.6 (green curve). However, the proposed scheme with overlap ratio 0.8 (green curve) significantly outperforms the proposed scheme with overlap ratio 0.7 (orange curve) and that with 0.6 (black curve). Thus, a training sample will be labelled as a positive sample (i.e., high tracking accuracy) if *O_r_* is greater than 0.8 and otherwise it will be labelled as a negative sample (i.e., non-high tracking accuracy). The training dataset is adopted for analyses of conditional probability distribution and training of the projection matrix of dimensionality reduction.

**Figure 9:**
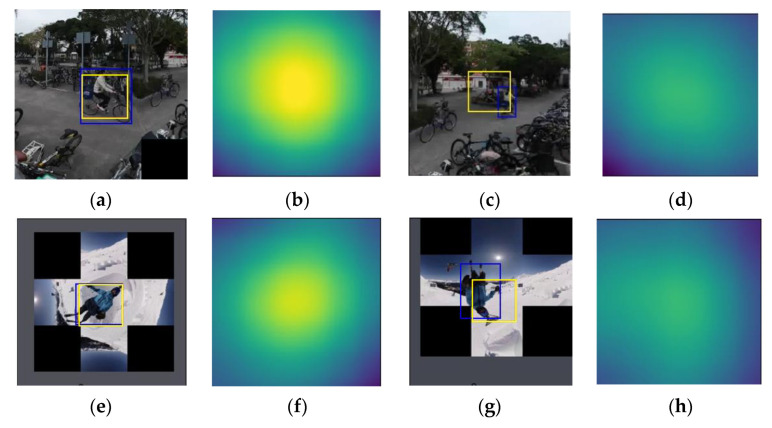
Tracking results (yellow bounding box) of SiamFC [12], the ground truth of the target (blue bounding box), and local window centering at the highest score on the score map. (**a**) Tracking result and ground truth of the 12th frame of video Ping-chou Halfday Tour [30], captured by a static camera. The overlap ratio is 0.74. (**b**) The score map of (**a**). (**c**) Tracking result and ground truth of the 80th frame of the video Ping-chou Halfday Tour, captured by a static camera. The overlap ratio is 0.14. (**d**) The score map of (**c**). (**e**) Tracking result and ground truth of the 4th frame of video Skiing [31], captured by a moving camera. The overlap ratio is 0.89. (**f**) The score map of (**e**). (**g**) Tracking result and ground truth of the 40th frame of the video Skiing [31], captured by a moving camera. The overlap ratio is 0.45. (**h**) The score map of (**g**).

**Figure 10:**
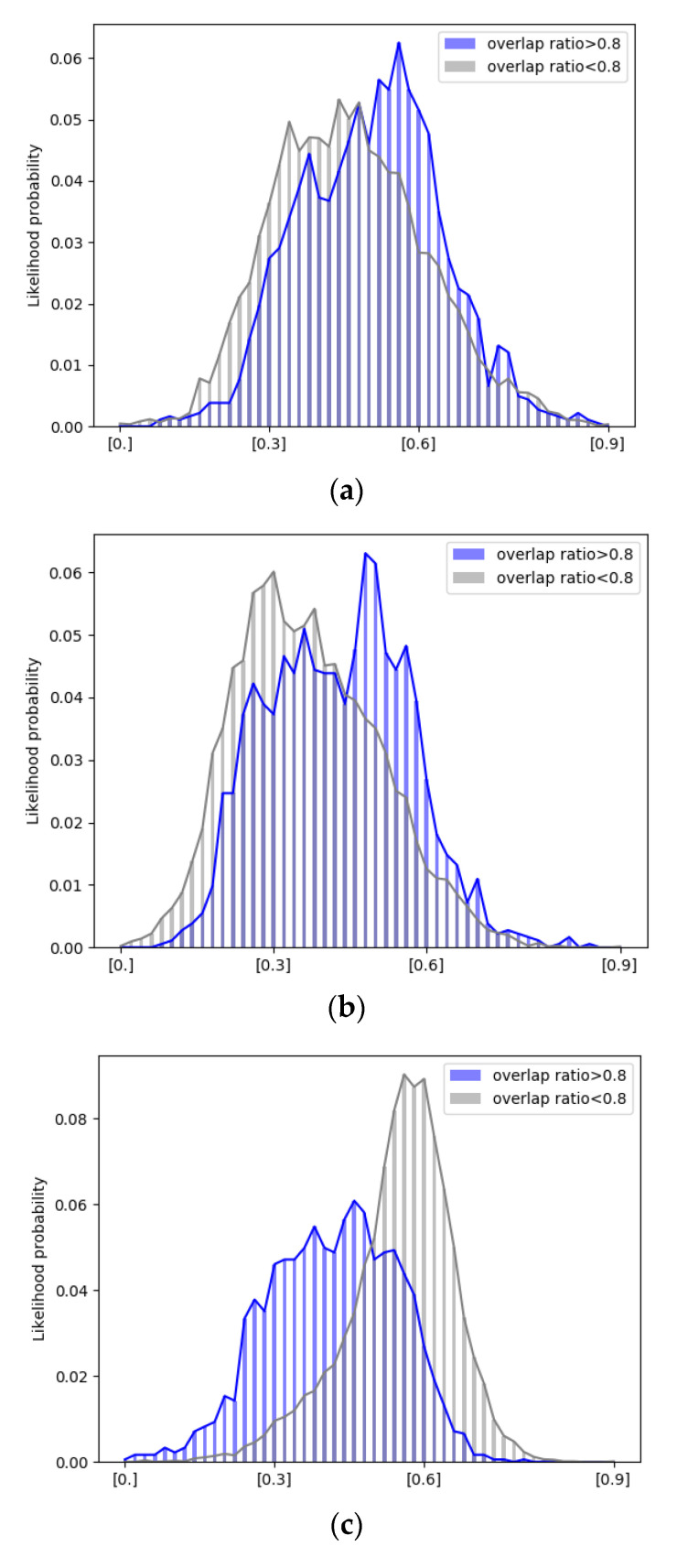
The likelihood probability distribution of a score map-based feature given high (blue)/non-high (grey) overlap ratio and clustering of training data. (**a**) Mean. (**b**) Standard deviation. (**c**) Fisher linear discriminant (FLD)-based feature (linear discriminant feature).

A score value on the score map of a fully convolutional Siamese networks represents similarity between the template and a candidate proposal that centers at the location in the search window. Figure 9 shows examples of tracking results of stitched images of EAC format and their corresponding score maps. Bounding boxes (i.e., tracking results) in Figure 9 have overlap ratios 0.74 and 0.89, respectively, and their corresponding score maps shown in Figure 9b,f both feature with high mean and high standard deviation. On the other hand, bounding boxes (i.e., tracking results) in Figure 9c,g have overlap ratios 0.14 and 0.45, respectively, and the corresponding score maps shown in Figure 9d,h both feature low mean and low standard deviation. That is, content characteristics of a score map are related to tracking accuracy in terms of overlap ratio. Thus, conditional probability mass functions (PMFs) of features (i.e., mean (Figure 10a) and standard deviation (Figure 10b) of the score map given high overlap ratio and those of features given non-high overlap ratio are analyzed. In Figure 10, the value of each feature is normalized to range between 0 and 1. Although the range of the feature value (e.g., mean) with high overlap ratio is similar to that of a feature with non-high overlap ratio, modes of these two groups distinctly separate from each other. In the meantime, the mode of the class with high overlap ratio has high mean (Figure 10a) and high standard deviation of scores (Figure 10b). Thus, it can be inferred that a probabilistic-based classifier is proper to classify an input sample that is represented by features of a score map.

In addition to statistics including mean and standard deviation of the score map providing clues to reflect tracking accuracy, this paper proposes to explore the discriminant feature of a score map by machine learning-based dimensionality reduction for binary classification of tracking accuracy. Dimensionality reduction transforms input data in high dimensional space to a low dimensional space without loss of information and aids in reducing the effect of outliers of the data [32]. Dimensionality reduction can be realized using either deep learning-based methods or non-deep learning-based methods. Considering dimension reduction and statistical optimization, this paper proposes to adopt a non-deep learning-based method, Fisher linear discriminant (FLD) [25], to explore the discriminant feature of a score map. Examples of non-deep learning-based methods are principle component analysis (PCA), independent component analysis (ICA), t-distributed stochastic neighbor embedding (t-sne), and Fisher linear discriminant (FLD) [25]. FLD was proposed by Ronald Aylmer Fisher in 1936 [25]. It is a supervised learning method used in machine learning, pattern recognition, and statistics. It aims at estimating the projection matrix from the training dataset off-line to well separate different classes. For two Gaussian classes where the covariance of one class is a multiple of the other, the Fisher discriminant yields the optimal linear discriminant. Otherwise, FLD produces a linear discriminant that optimizes the weighted classification error [33]. The objective function focuses on minimization of the within-class scatter matrix and maximization of between-class scatter matrix of training samples. In the test phase, the FLD-based feature vector of an input sample $X^{\prime }$ can be computed by projecting the data $X^{\prime }$ onto the projection matrix. The *n* dimensional data $X^{\prime }$ is projected onto the *m* dimensional data $Y^{\prime }$ by the projection matrix $\hat{P}$ with m<n.

(15) $$Y^{\prime }={\hat{P}}^{T}X^{\prime }$$

In the training phase of FLD, the divergence of training samples is measured by scatter matrices. Assume there are *L* classes in the training dataset. For training data $\hat{X}$, the within-class scatter matrix $S_{w}$ is defined by [29]

(16) $$S_{w}=\overset{L}{\underset{i=1}{\sum }}P_{i}E[\left(\hat{X}-M_{i}\right){\left(\hat{X}-M_{i}\right)}^{t}|\omega_{i}]$$

where $P_{i}$ is the prior probability of the *i*th class, and $M_{i}$ is the mean vector of the *i*th class, *ω_i_*. The between-class scatter matrix $S_{b}$ is defined by

(17) $$S_{b}=\overset{L}{\underset{i=1}{\sum }}P_{i}\left(M_{i}-M_{o}\right){\left(M_{i}-M_{o}\right)}^{t}$$

where $M_{o}$ is the mean vector of all training samples. The divergence of all training samples, mixture scatter matrix, is equal to the sum of $S_{w}$ and $S_{b}$

(18) $$S_{M}=E[\left(\hat{X}-M_{o}\right)\left(\hat{X}-M_{o}{)}^{t}\right]=S_{w}+S_{b}$$

where *m* is less than *n*. Thus, the between-class scatter matrix of the input data after dimensionality reduction $\hat{Y}$ of FLD is computed by

(19) $$S_{b\hat{Y}}=\overset{L}{\underset{i=1}{\sum }}P_{i}\left({\hat{P}}^{t}M_{i}-{\hat{P}}^{t}M_{o}\right){\left({\hat{P}}^{t}M_{i}-{\hat{P}}^{t}M_{o}\right)}^{t}={\hat{P}}^{t}S_{b}\hat{P}$$

In addition, the within-class scatter matrix of $\hat{Y}$ is computed by

(20) $$S_{w\hat{Y}}={\hat{P}}^{t}S_{w}\hat{P}$$

Thus, the training phase of FLD is to find ${\hat{P}}_{opt}$ that maximizes the objective function $tr\left(S_{w\hat{Y}}^{-1}S_{b\hat{Y}}\right)$

(21) $${\hat{P}}_{opt}=arg\;\underset{\hat{P}}{max}\;tr\left(S_{W\hat{Y}}^{-1}S_{b\hat{Y}}\right)$$

where ${\hat{P}}_{opt}$ satisfies

(22) $$\left(S_{W}^{-1}S_{b}\right){\hat{P}}_{opt}={\hat{P}}_{opt}\left(S_{W\hat{Y}}^{-1}S_{b\hat{Y}}\right)$$

By diagonalizing $S_{w\hat{Y}}$ and $S_{b\hat{Y}}$,

(23) $$B^{T}S_{b\hat{Y}}B=U_{m}$$

(24) $$B^{T}S_{w\hat{Y}}B=I_{m}$$

where *U_m_* and $I_{m}$ are diagonal matrices, and *B* is a m×m square matrix that is invertible. By introducing Equations (23) and (24) into Equation (22),

(25) $$\left(S_{W}^{-1}S_{b}\right){\hat{P}}_{opt}B=\left({\hat{P}}_{opt}B\right)U_{m}$$

Finally, the optimal projection matrix ${\hat{P}}_{opt}$ is constructed from the first *m* eigenvectors that correspond to the first *m* greatest eigenvalues. In the test phase of FLD, the optimal projection matrix ${\hat{P}}_{opt}$ reduces an input sample (e.g., a score map) in the *n*-dimensional space to the *m*-dimensional space.

**Figure 11:**
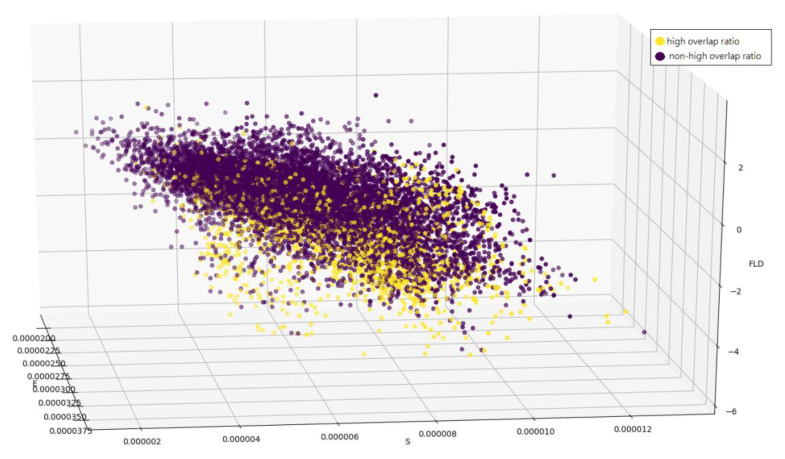
A cluster of training samples with high overlap ratio (yellow dot) and that with non-high overlap ratio (purple dot) in the 3-D feature (linear discriminant feature, mean, and standard deviation) space.

It is noticed that if there are insufficient training samples, FLD will fail due to the problem of small sample space (SSS) [29]. In addition, the n-dimensional data with L classes can be reduced to at most L−1 dimensions by FLD given that n is greater than L−1 [29]. In the proposed detector of tracking results with high overlap ratio, *n* is 10,000 and L is 2 (i.e., class of tracking results of high overlap ratio and class of tracking results of non-high overlap ratio). Accordingly, each sample (i.e., score map) in our proposed scheme has a one-dimensional FLD-based feature with the aid of a projection matrix that is trained off-line. Figure 11 shows conditional probability mass functions (PMFs) of the FLD-based feature using 10,407 training samples. The conditional PMM when the score map has high overlap ratio differs from the conditional PMF when the score map has non-high overlap ratio. A score map with a lower feature value (e.g., 0.4) has higher probability to have high overlap ratio. Thus, each score map can be represented by a three-dimensional feature vector that consists of the mean of a score map, the standard deviation of a score map, and an FLD-based feature of a score map. Figure 11 shows two clusters of 10,407 training samples, where a yellow dot denotes a sample with high overlap ratio and a purple dot denotes a sample with non-high overlap ratio.

### 3.3. The Proposed Machine Learning Based Timing Detector of Template Update

By referring to conditional probability distributions of features of a score map of SiamFC (Section 3.2), it is reasonable to adopt a probabilistic-based classifier to decide the timing of template update where the input sample is represented by features of a score map. Thus, this subsection proposes to adopt the optimum statistical classifier, the Bayes classifier, for determination of update timing. In the following, an overview of the Bayes classifier is provided. Then the proposed Bayes classifier-based timing detector of template update for the fully convolutional Siamese networks-based tracker is stated.

The Bayes classifier predicts the label of an input sample by referring to the pre-trained likelihood distributions $p(X|\omega_{k})$ and prior distributions (i.e., the occurrence of the kth class) $p\left(\omega_{k}\right)$, where *X* is input data, *ω_k_* is the kth class, and k ranges from 1 to W. The Bayes classifier aims at minimization of the total average loss [24]

(26) $$r_{j}\left(X\right)=\sum_{k=1}^{W}L_{kj}\left(X\right)p(X|\omega_{k})p\left(\omega_{k}\right)/p\left(X\right)$$

where $L_{kj}\left(X\right)$ is the loss caused by assigning the input sample *X* to the *j*th class while *X* belongs to the *k*th class. $p(\omega_{k}|X)$ is the posterior probability that *X* comes from $\omega_{k}$.

For binary classification (i.e., *W* = 2) of our proposed scheme, $\omega_{0}$ and $\omega_{1}$ are the class with non-high overlap ratio and the class with high overlap ratio, respectively. The input data $X={\left[x_{M},x_{s},x_{F}\right]}^{t}$, where $x_{M}\in \left[0,1\right]$ is a n×n sub-image of the mean of a score map, $x_{s}\in \left[0,1\right]$ is the standard deviation of the n×n sub-image of a score map, and $x_{F}\in \left[0,1\right]$ is Fisher linear discriminant-based 1-D feature of an n×n sub-image of a score map, where the unnormalized value of $x_{F}$ ranges from −5.6 to 2.4. $p(X|\omega_{0})$ is the likelihood of the data given that *X* belongs to the class with high overlap ratio, and $p(X|\omega_{1})$ is the likelihood of the data given that *X* belongs to the class with non-high overlap ratio. Both $p(X|\omega_{0})$ and $p(X|\omega_{1})$ are learned off-line. Since there will be no loss if the label of *X* and the predicted result of the classifier are the same, $L_{00}\left(X\right)=0$, $L_{01}\left(X\right)=1$, $L_{10}\left(X\right)=1$, and $L_{11}\left(X\right)=0$ in tests. In the test phase of the proposed detector of update timing, the predicted result *Y* is

(27) $$Y=\{\begin{array}{ccc} 0 & if & L_{01}\left(X\right)p(X|\omega_{0})p\left(\omega_{0}\right)<L_{10}\left(X\right)p(X|\omega_{1})p\left(\omega_{1}\right).\\ 1 & & otherwise. \end{array}$$

That is, if $r_{1}\left(X\right)$ is less than $r_{0}\left(X\right)$, the predicted result of *X* will be 1. That is, the score map represented by the feature vector *X* will be taken as having non-high overlap ratio. Thus, the template should not be updated since the tracking result is inaccurate. If $r_{1}\left(X\right)$ is greater than $r_{0}\left(X\right)$, the predicted result will be 0. It suggests that the template can be updated since tracking accuracy is high. Since p(X) does not vary with $r_{j}\left(X\right)$, it can be ignored during comparisons of decision functions of two classes.

In the tracking phase, the fully convolutional Siamese networks-based tracker SiamFC computes the score map using the template and the candidate proposal extracted from the search window. For the sub-image of the score map, the feature vector that consists of the mean, the standard deviation, and the Fisher linear discriminant-based feature extracted by the projection direction is constructed. By referring to the feature vector, the Bayes classifier-based timing detector of template update decides whether the original template is replaced by the estimated bounding box of the current frame or not. To the avoid template drifting problem caused by frequent update, the duration between two updates is constrained to be not less than $T_{P}$ frames. If the timing detector of template update suggests that the template can be updated after prediction of the bounding box in the *t*th frame and there is no template update from the (*t* − ($T_{P}-1$))th frame to the *t*th frame, the proposed tracker will take the action of template update.

## 4. Experimental Results

The proposed scheme is realized by revising the implementation of SiamFC [34]. Test results are divided into five parts. The first part (Section 4.1) provides performance analyses of individual components of the proposed scheme. Comparisons among SiamFC [12], the original SiamFC with face stitching proposed in [23], and our proposed scheme are made. The second part (Section 4.2) compares the proposed scheme with the only existing tracker designed for 360-degree EAC format videos, proposed by Tai et al. [23]. Since the focus of the proposed scheme is the score map-based timing detector of template update, the third part (Section 4.3) compares the proposed timing detector with a state of the art score map-based timing detector of template update [15,21]. For this, the authors implement the timing detector APCE (Section 1.3) which is proposed by Wang et al. and is adopted by LSSiam (Section 1.3) with the baseline of the Siamese networks-based tracker SiamFC [12,15,21]. The fourth part (Section 4.4) validates the effectiveness of the proposed template update scheme for Siamese networks-based trackers on 360-degree EAC format videos. The Siamese networks-based tracker SA-Siam [16] is revised to integrate face stitching and the proposed timing detector of template update. The fifth part provides analyses of time complexity of all considered trackers in Section 4. In addition, the execution time of individual components of the proposed scheme is analyzed.

**Figure 12:**
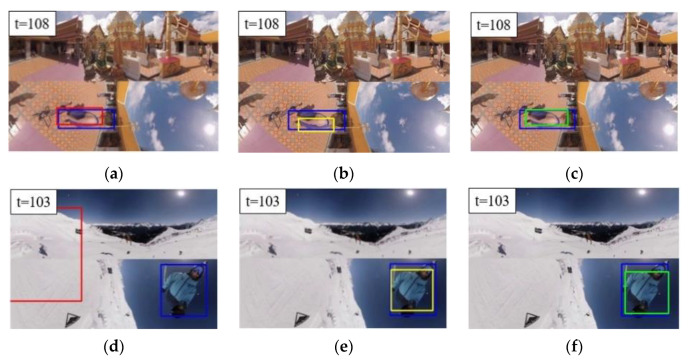
Tracking results of targets located in regions with significant geometric deformation of videos #4 and #8. (Blue: ground truth. Red: SiamFC + face stitching (S). Green: SiamFC + S + template update (P). Yellow: SiamFC + S + H) (**a**) SiamFC + S on video #4. (**b**) Tai et al. (SiamFC + S + H) on video #4. (**c**) Proposed scheme (SiamFC + S + P) on video #4. (**d**) SiamFC + S on video #8. (**e**) Tai et al. (SiamFC + S + H) on video #8. (**f**) Proposed scheme (SiamFC + S + P) on video #8.

To validate the effectiveness of the proposed scheme, the training dataset of the Fisher linear discriminant and the Bayes classifier-based timing detector of the proposed scheme does not overlap with the test dataset of the proposed tracking scheme. SiamFC is trained using the VID dataset of the ILSVRC2015 dataset that consists of a variety of videos with more than one million frames [26]. Implementation details are as suggested in [12]. The training dataset of the Fisher linear discriminant and the Bayes classifier-based timing detector of the proposed scheme is generated using 21 single-view videos in [28], as described in Section 3. The test dataset consists of eight 360-degree EAC format videos with a variety of test conditions, including Spotlight (video #1) [31], London on Tower Bridge (video #2) [7], London Park Ducks and Swans (video #3) [7], Doi Suthep (video #4) [7], Amsterdam (video #5) [31], Paris-view6 (video #6) [31], Ping-Chou Half-day Tour (video #7) [30], and Skiing (video #8) [31]. Videos #1 and #8 are captured by moving cameras, and videos #2-#7 are captured by static cameras. Except video #6 that is captured at 60 fps, the frame rate of each test video is 30 fps. Videos #1 and #6 feature illumination variations. Significant geometric deformation of targets occurs in videos #4 and #8 (Figure 12). The target (i.e., a skier) has a complex motion model (e.g., in-plane rotation) in video #8. The target in video #6 is occluded for a period of time. The target moves across different faces in all test videos. Videos are downsampled before tracking, where the spatial resolution of one downsampled face of videos #1–#6 and #8 is 144×144, and that of video #7 is 588×588. The simulation environment is stated as follows. The CPU (Central Processing Unit) and GPU (Graphics Processing Unit) are i7-7700K 4.5GHz and GeForce GTX 1080 Ti, respectively. RAM is DDR4(48G). The OS (Operating System) is Ubuntu 18.04-x64. Tensorflow with Python 3.5 is adopted.

### 4.1. Performance Analyses of Individual Components of the Proposed Scheme

Table 2 provides comparisons of tracking accuracy in terms of average overlap ratio and average location error among the original SiamFC [12], SiamFC with face stitching (SiamFC + S), and our proposed machine learning-based scheme (SiamFC + S + P). Test results show that face stitching helps existing trackers (e.g., SiamFC) in the face of content discontinuity between several faces of EAC format. The reason is that there is a significant gap between location error and overlap ratio of SiamFC and those of SiamFC + S for most of the test videos, respectively. Moreover, the proposed timing detector of template update (SiamFC + S + P) further improves the tracking accuracy of SiamFC + S on videos #4-#8. Tracking accuracy of the proposed scheme (SiamFC + S+P) is similar to that of SiamFC + S on videos #1 and #2 due to low update frequency of the proposed scheme, where the proposed scheme achieves a higher overlap ratio on videos #1 and #2 while SiamFC + S leads to a smaller location error on these two videos (Table 2). The target in video #3 features with slow motion and slight deformation such that SiamFC + S can track the target well. However, the proposed scheme with template update easily leads to a slight drifting problem and thus SiamFC + S slightly outperforms the proposed scheme (Table 3). Figure 12 provides tracking results of targets located in regions with significant geometric deformation of videos #4 and #8. For the 108th frame of video #4, the proposed scheme (Figure 12c) outperforms SiamFC + S (Figure 12a) and Tai et al. (SiamFC + S + H) (Figure 12b). For the 103th frame of video #8, both the proposed scheme (Figure 12f) and Tai et al. (SiamFC + S + H) (Figure 12e) significantly outperform SiamFC + S (Figure 12d).

**Figure 13:**
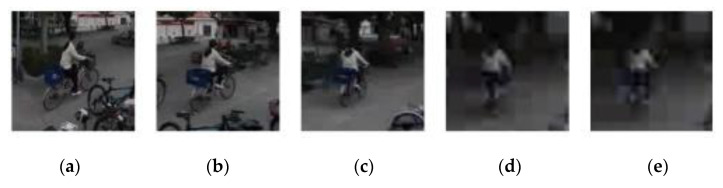
Templates selected by the proposed scheme (video #7). (**a**) The 35th frame. (**b**) The 58th frame. (**c**) The 93rd frame. (**d**) The 177th frame. (**e**) The 187th frame.

**Figure 14:**
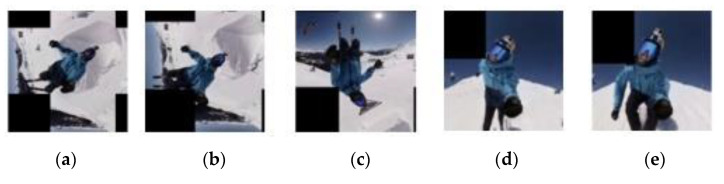
Templates selected by the proposed scheme (video #8). (**a**) The 10th frame. (**b**) The 25th frame. (**c**) The 38th frame. (**d**) The 166th frame. (**e**) The 180th frame.

By cropping from 1592 score maps that are calculated using SiamFC with face stitching and the proposed timing detector (SiamFC + S + P) on the test dataset, 1592 sub-images are generated. A total of 1496 test samples have negative labels and 96 samples have positive labels. Test results show that accuracy of the proposed timing detector approaches 90%, where there are 7 true positives, 89 false negatives, 75 false positives, and 1421 true negatives (Table 4). By referring to the ground truth, a sample will have a positive label if the tracking result of SiamFC + S + P has an overlap ratio that is greater than 0.8. Thus, 6% (i.e., 96/1592) of predicted bounding boxes (i.e., tracking results) using SiamFC + S + P have high overlap ratio. If a smaller threshold (e.g., 0.6) of overlap ratio is selected, the number of positive samples will increase, and the imbalance problem will be solved. However, template update with an inaccurate new template (lower overlap ratio) easily decreases tracking accuracy. The numbers of template updates of videos #1–#8 are 1, 4, 12, 10, 7, 3, 5, 5, respectively. It is noted that misclassification of a positive sample (i.e., the ground truth has high overlap ratio) causes the right timing to be missed while frequent update leads to the drifting problem. For the proposed timing detector with a true positive rate of 1.5%, low update frequency will avoid a worse one if visual features of the target change slightly over time. For targets with time-varying features, infrequent update with an accurate template still contributes to improvement of tracking accuracy (e.g., videos #7 and #8 (Figure 13 and Figure 14). On the contrary, misclassification of a negative sample (i.e., the ground truth has non-high overlap ratio) leads to the original template being replaced by an inaccurate template and thus the drifting problem arises. However, the proposed timing detector has low false positive rate 3.7% as expected.

**Figure 15:**
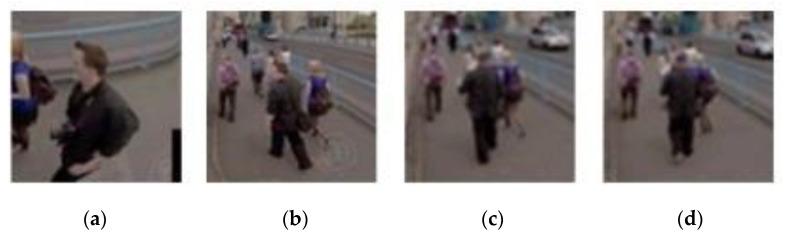
Templates selected by the proposed scheme (video #2). (**a**) The 43rd frame. (**b**) The 131st frame. (**c**) The 186th frame. (**d**) The 194th frame.

**Figure 16:**
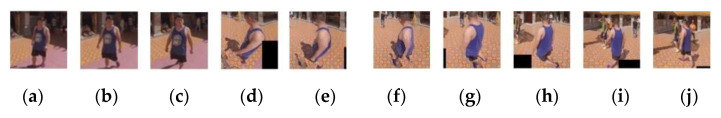
Templates selected by the proposed scheme (video #4). (**a**) The 22nd frame. (**b**) The 33rd frame. (**c**) The 48th frame. (**d**) The 93rd frame. (**e**) The 101st frame. (**f**) The 109th frame. (**g**) The 118th frame. (**h**) The 134th frame. (**i**) The 158th frame. (**j**) The 183rd frame.

Templates selected by the proposed scheme are visually inspected in Figure 13, Figure 14, Figure 15 and Figure 16. It is noticed that SiamFC constructs a template by adding a margin to the scaled bounding box for context [12]. Regardless of the number of updates, most templates selected by the proposed timing detector have a high overlap ratio with the ground truth, except those of video #6. Tracking on video #6 is challenging. It has a cluttered background, and deformation and illumination variation occur after the target is fully occluded for a period of time (Table 5). However, template update with the proposed timing detector still slightly improves tracking accuracy of SiamFC + S on video #6 (Table 5). SiamFC + S on video #7 has an average overlap ratio of 0.236 and an average location error of 90.486. The proposed template update scheme (SiamFC + S + P) significantly improves tracking accuracy in terms of average overlap ratio 0.593 and average location error 9.264 (Table 2). The proposed scheme (SiamFC + S + P) also outperforms SiamFC + S on video #8. The reason is that visual features of the target vary over time notably while the target (i.e., a skier) has a complex motion model. SiamFC+S has an average overlap ratio of 0.235 and an average location error of 140.250 while the proposed scheme with the aid of template update has an average overlap ratio of 0.518 and an average location error of 27.011 (Table 2).

### 4.2. Comparisons with State-of-the-Art People Trackers for 360-Degree EAC Format Videos

The only existing visual tracker designed for 360-degree EAC format videos is proposed by Tai et al. (SiamFC + S + H) [23], where the heuristics-based timing detector was proposed. Thus, Table 6 provides comparisons of overlap ratio and location error between SiamFC + S + H and our proposed machine learning-based scheme (SiamFC + S + P).

**Figure 17:**
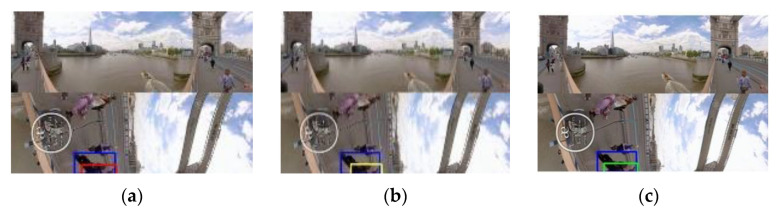

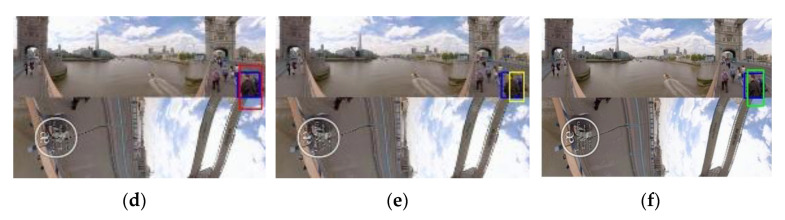
Samples of the predicted bounding box in the face of non-uniform deformation and occlusion caused by padded pixels on video #2. (Blue: ground truth) (**a**) SiamFC + S, the 25th frame. (**b**) SiamFC + S + H (Tai et al.) [23], the 25th frame. (**c**) Proposed scheme, the 25th frame. (**d**) SiamFC + S, the 104th frame. (**e**) SiamFC + S + H (Tai et al.) [23], the 104th frame. (**f**) Proposed scheme, the 104th frame.

**Figure 18:**
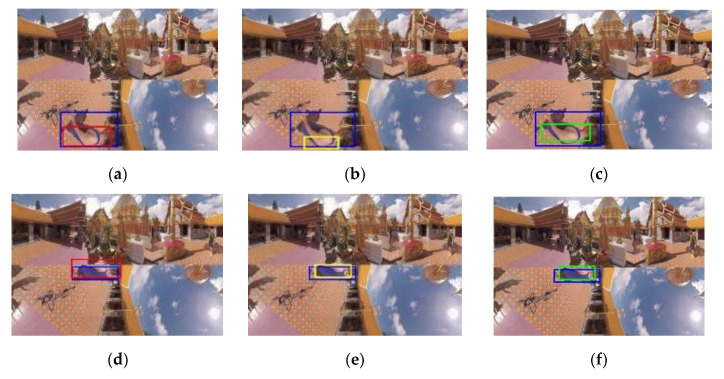
Samples of predicted bounding box in the face of non-uniform deformation and occlusion caused by padded pixels on video #4. (Blue: ground truth) (**a**) SiamFC + S, the 96th frame. (**b**) SiamFC + S + H (Tai et al.) [23], the 96th frame. (**c**) Proposed scheme, the 96th frame. (**d**) SiamFC + S, the 142th frame. (**e**) SiamFC + S + H (Tai et al.) [23], the 142th frame. (**f**) Proposed scheme, the 142th frame.

Although SiamFC + S + H heuristically selects thresholds of mean and standard deviation for each test video [23], our proposed machine learning-based method (SiamFC + S + P) still outperforms SiamFC + S + H on videos #2, #3, #4, and #6 without heuristic settings of parameters. For video #1, tracking accuracy of SiamFC + S + P is similar to that of SiamFC + S + H. For video #5, although tracking accuracy of SiamFC + S + P (the proposed scheme) is slightly lower than that of SiamFC + S + H (Tai et al.) [23], tracking results of most frames of these two trackers are similar. Moreover, there is no difference between the predicted bounding box of proposed scheme and that of Tai et al. on the last frame of Video #5 (Table 7). For videos #7 and #8, SiamFC + S + P still significantly improves tracking accuracy of SiamFC + S; even SiamFC + S + P does not outperform SiamFC + S + H on videos #7 and #8. Figure 17 and Figure 18 provide comparisons of tracking results of SiamFC + S, SiamFC + S + H (Tai et al.) [23], and SiamFC + S + P (the proposed scheme) on frames of videos #2 and #4 with combination of non-uniform geometric deformation and occlusion, and fast motion as the target moves into the boundary of a face of EAC format. The face stitching scheme indeed helps the tracker solve the problem of content discontinuity between faces, and the proposed timing detector of template update selects the right timing such that the tracker can successfully adapt to the severe change of the target’s appearance at a short notice.

### 4.3. Comparisons with State-of-the-Art Score Map Based Timing Detector of Template Update

**Figure 19:**
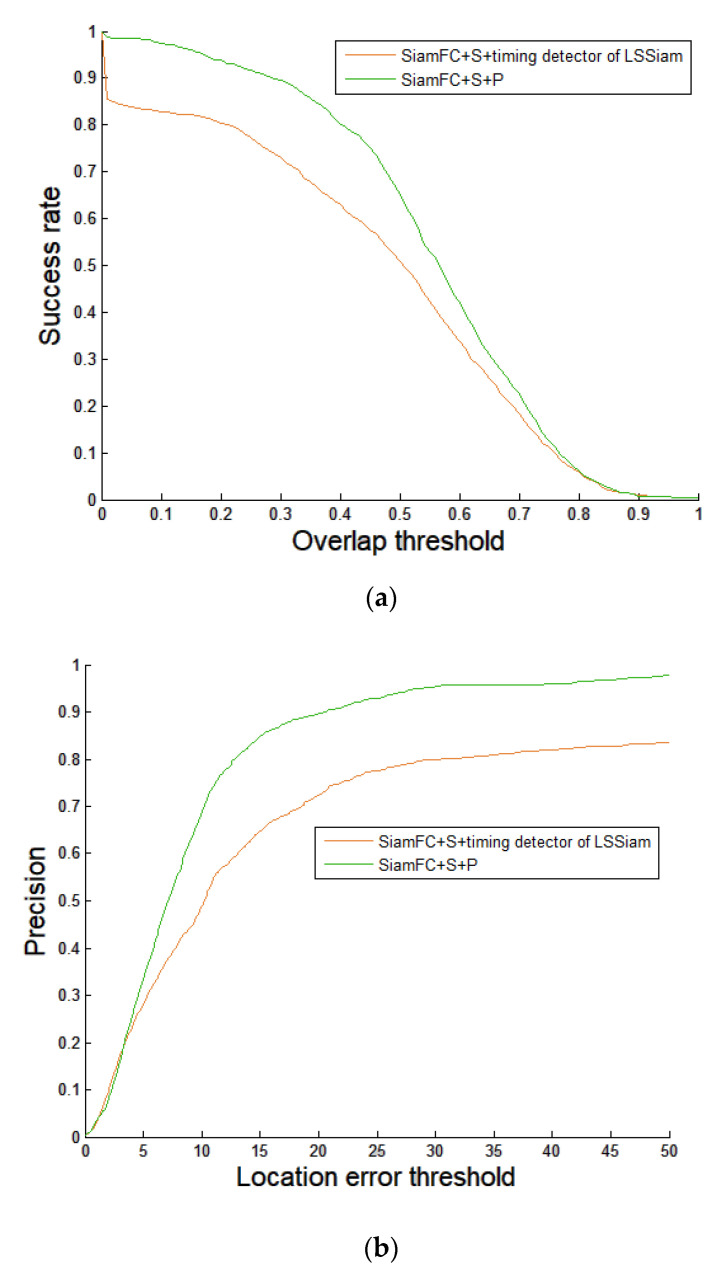
Comparisons between SiamFC + S +Timing detector of template update of LSSiam (proposed by Liang et al.) and the proposed scheme (SiamFC + S + P). Eight test videos are tested. (**a**) Success plots. (**b**) Precision plots.

Only a few schemes have proposed Siamese networks-based timing detectors of template update. The authors implement the timing detector APCE that was proposed by Wang et al. and was adopted by LSSiam (Section 1.3.) [15,21]. In our implementation of the timing detector of LSSiam [10], the template will be updated if both of two conditions are satisfied. (1) The confidence score is greater than six. (2) Frame interval between two updates is not less than 40 frames, as suggested in [15]. Since the focus of our proposed scheme is how to detect the right timing instead of how to generate a new template, fair comparisons between our proposed timing detector and that of LSSiam are made using the same naive new template (i.e., the predicted bounding box at the time instant that is selected). Performance evaluation in terms of precision plot and success plot of the proposed scheme and SiamFC + S + Timing detector of template update of LSSiam on the test dataset is shown in Figure 19a,b, respectively. The success plot is generated using the overlap ratio between the predicted bounding box and the ground truth. If the overlap ratio is greater than a given threshold, the target will be taken as being tracked successfully in one frame. In the success plot, the success rate represents the ratios of successful frames at different thresholds that range between zero and one. The precision plot is generated using the Euclidean distance-based location error between the center of the predicted bounding box and that of the ground truth. The precision rate indicates the ratio of frames with location error that is less than a given threshold [28]. As we can see, the proposed scheme (SiamFC + S + P) outperforms SiamFC with face stitching and the timing detector of LSSiam (SiamFC + S +Timing detector of LSSiam) on both precision plots and success plots. The numbers of template updates of LSSiam + S on videos #1–#8 are 3, 4, 4, 4, 4, 4, 4, 0, 2, respectively.

### 4.4. Validation of the Effectiveness of the Proposed Template Update Scheme for Siamese Networks Based Tracking on 360-Degree EAC Format Videos

**Figure 20:**
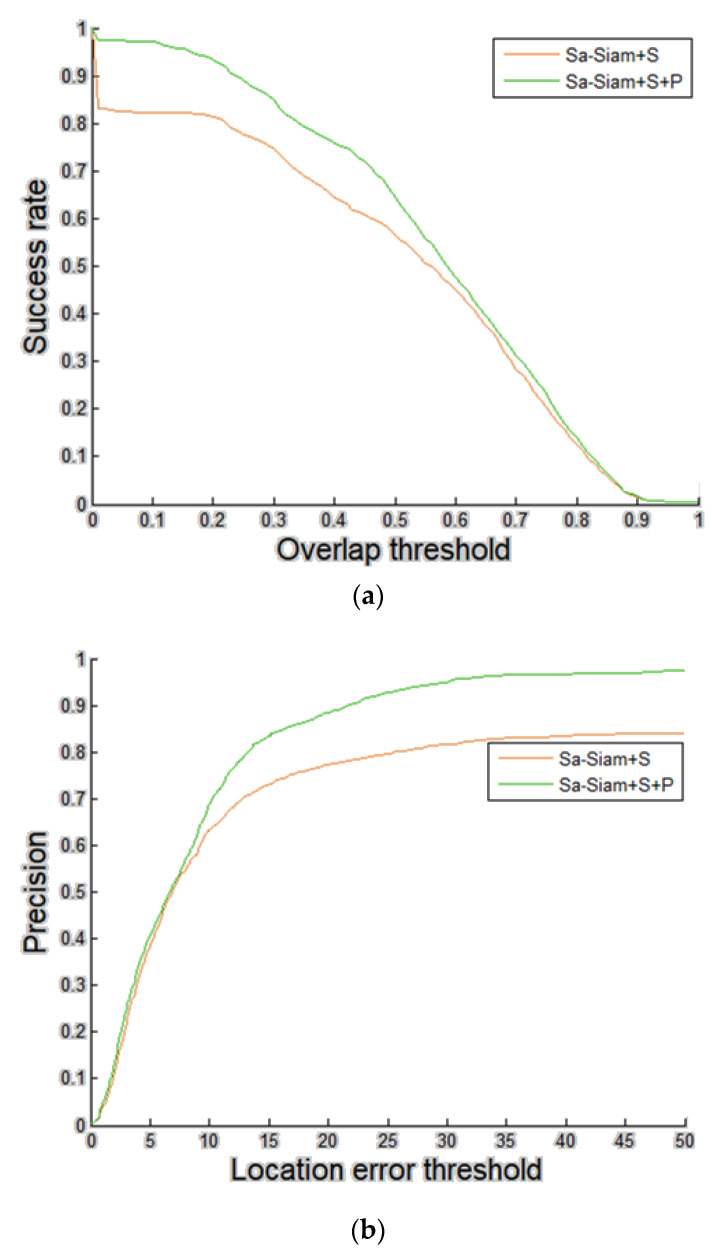
Comparisons between SA-Siam with face stitching (SA-Siam + S) and SA-Siam with face stitching and the timing detector of template update (SA-Siam + S + P). Eight test videos with 1600 frames were tested. (**a**) Success plots. (**b**) Precision plots.

To validate the effectiveness of the proposed template update scheme for tracking on 360-degree EAC format videos, face stitching and a Bayes classifier-based timing detector of template update, referring to a Fisher Linear Discriminant (FLD)-based feature and statistical feature of a sub-image of a score map and face stitching (Section 3), are incorporated into SA-Siam [16]. The training dataset of FLD and the Bayes classifier is constructed using sub-images of overall score maps’ output by SA-Siam. Comparison of tracking accuracy between the SA-Siam with face stitching (SA-Siam + S) and SA-Siam combined with face stitching and the proposed timing detector (SA-Siam + S + P) is shown in Figure 20. The average overlap ratios of the SA-Siam + S and SA-Siam + S+P are 0.4884 and 0.5587, respectively. The average location errors of the SA-Siam + S and SA-Siam +S + P are 36.7006 and 9.8804, respectively. As we can see, the timing detector of template update significantly improves the tracking accuracy of SA-Siam. The number of template updates of SA-Siam + S on videos #1–#8 is 8, 1, 4, 0, 0, 2, 5, 14, respectively.

**Figure 21:**
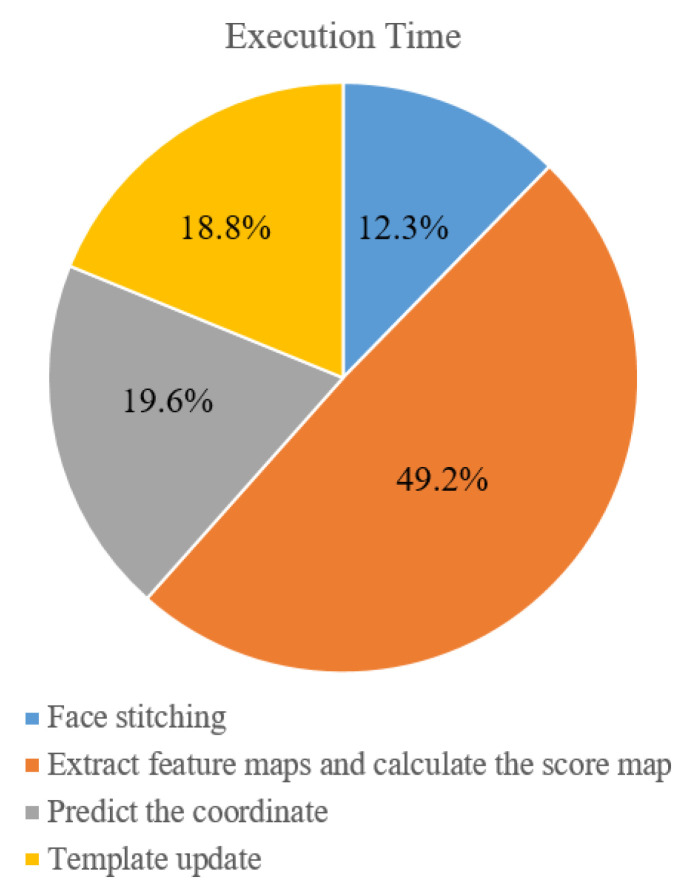
The percentage of computational time of individual components of the proposed scheme including face stitching, extraction of feature maps and calculation of the score map, prediction of the coordinate, and template update.

Comparisons of the tracking speed of considered trackers in Section 4. are shown in Table 8. For videos #1–#6 and #8, the tracking speed of the proposed tracker is 52.9 fps (beyond the acquisition rate). For video #7 which is not downsampled before tracking, the tracking speed of the proposed tracker is 11.2 fps. Although the tracking speed of the proposed scheme is slower than those of SiamFC with face stitching and the timing detector of template update of LSSiam and Tai et al. (SiamFC + S + H), the proposed scheme achieves the highest tracking accuracy among the compared trackers (Section 4.3). The percentage of computational time of components of the proposed scheme is further analyzed in Figure 21. Siamese networks-based prediction occupies the largest portion (49.2%) of the exeuction time while face stitching occupies the smallest portion (12.3%) of the execution time.

## 5. Conclusions

This paper proposes a Siamese networks-based tracker using template update and face stitching for 360-degree EAC format videos. The proposed scheme overcomes the problem of content discontinuity of the EAC format and enables fast speed (52.9 fps). The reasons why the proposed tracker operates beyond the acquisition rate are stated as follows. (1) The Siamese networks-based tracker enables fast speed since it has a small number of convolutional layers and simple operation of similarity. (2) Face stitching does not apply coordinate conversion between the EAC format and the sphere domain. (3) The Fisher linear discriminant and statistics of a score map are computed with low computation complexity. (4) The Bayes classifier-based timing detector of template update is computed with low computation complexity. Finally, with the aid of the proposed machine learning-based timing detector of template update that refers to the linear discriminant and statistical features of a score map, the proposed tracker can adapt to notable change of the target appearance over time. Future work will focus on design of a deep learning-based timing detector of template update.

## Figures and Tables

**Table 1:** Summarization of described trackers in Section 1.

	Examples	Template Update	Timing Detector of Template Update	Year	Remark
**Trackers for 360-Degree Videos**	Liu et al. 2018 [4]	X	X	2018	1. ERP format 2. DeepSort-based [5]
	Zhou et al. [6]	X	X	2010	1. CMP format 2. Mean Shift-Based
**The First Deep Learning-Based Tracker**	DLT [9]	X	X	2013	Stacked autoencoder-based
**Siamese Networks Based Trackers**	GoTurn [11]	X	X	2016	Pioneer
	SiamFC [12]	X	X	2016	Pioneer
	SiamRPN [13]	X	X	2018	Region proposal network
	SiamRPN++ [14]	X	X	2019	Effective sampling strategy
	SA-Siam [16]	X	X	2018	Semantic branch and similarity branch
	adaDCF [17]	X	X	2020	FDA discriminates between foreground and background
	CFNet [18]	Aggressive strategy	X	2017	Correlation filter-based template generation
	Yang et al. [19]	Aggressive strategy	X	2018	Attentional LSTM controls memory for template generation
	Xu et al. [20]	Non-aggressive strategy	Using highest score of a score map	2019	UAVs-Based Tracking
	Wang et al. [21]	Non-aggressive strategy	APCE of a score map	2017	
	Liang et al. [15]	Non-aggressive strategy	Update interval and APCE [21]	2020	
	Tai et al. [23]	Non-aggressive strategy	Statistics of score map	2020	EAC format

**Table 2:** Comparisons of Tracking Accuracy among SiamFC [12], SiamFC + S, and SiamFC + S + P (proposed scheme) (S: Face Stitching. P: Template Update).

	Overlap Ratio			Location Error (Units: Pixels)		
	SiamFC	SiamFC + S	SiamFC + S + P (Proposed)	SiamFC	SiamFC + S	SiamFC + S + P (Proposed)
**Video#1**	0.046	0.481	0.479	106.343	10.171	10.152
**Video#2**	0.065	0.563	0.547	118.246	9958	9730
**Video#3**	0.121	0.728	0.688	94.033	2270	2714
**Video#4**	0.247	0.518	0.532	84.880	7507	6746
**Video#5**	0.607	0.648	0.653	6276	5936	4956
**Video#6**	0.303	0.363	0.366	25.024	11.341	10.763
**Video#7**	0.100	0.236	0.593	316.711	90.486	9264
**Video#8**	0.102	0.235	0.518	172.300	140.250	27.011
**Average**	0.199	0.491	0.547	115.477	34.740	10.167

**Table 3:** Samples of Tracking Results of SiamFC + S and SiamFC + S + P (proposed scheme) on Video #3 (London Park Ducks and Swans). The last frame index is 200. (S: Face Stitching. P: Template Update. Blue box: Ground Truth).

	t = 2	t = 120	t = 200
**SiamFC + S**	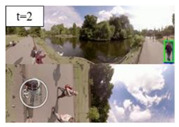	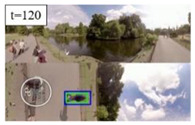	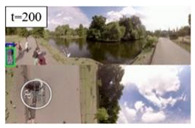
**SiamFC + S + P** **(Proposed Scheme)**	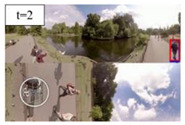	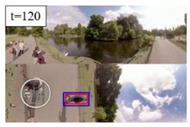	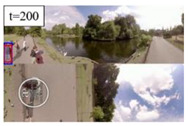

**Table 4:** Confusion matrix of the proposed timing detector.

		Ground Truth	
		True	False
**Prediction**	**Positive**	7	75
	**Negative**	89	1421

**Table 5:** Samples of tracking results of SiamFC + S and SiamFC + S + P (proposed scheme) on video #6 (Paris-view 6). The last frame index is 200. (S: Face Stitching. P: Template Update. Blue box: Ground Truth.)

	t = 2	t = 120	t = 200
**SiamFC + S**	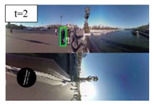	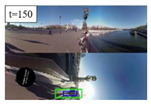	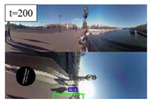
**SiamFC + S+P** **(Proposed Scheme)**	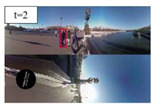	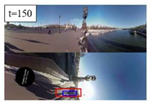	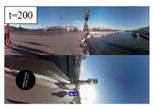

**Table 6:** Comparisons of tracking accuracy between SiamFC + S + H (Tai et al.) and SiamFC + S + P (proposed scheme). (S: Face Stitching. P: Template Update).

	Overlap Ratio		Location Error (Units: Pixels)	
	SiamFC + S + H (Tai et al.) [23]	SiamFC + S + P (Proposed)	SiamFC + S + H (Tai et al.) [23]	SiamFC + S + P (Proposed)
**Video#1**	0.481	0.479	10.160	10.152
**Video#2**	0.405	0.547	13.267	9730
**Video#3**	0.660	0.688	3718	2714
**Video#4**	0.531	0.532	7702	6746
**Video#5**	0.692	0.653	4043	4956
**Video#6**	0.337	0.366	11.928	10.763
**Video#7**	0.599	0.593	5758	9264
**Video#8**	0.572	0.518	17.330	27.011
**Average**	0.534	0.547	9238	10.167

**Table 7:** Samples of tracking results of SiamFC + S + H (Tai et al.) and SiamFC + S + P (proposed Scheme 5. (Amsterdam, The Netherlands). The last frame index is 200. (S: Face Stitching. P: Template Update. Blue box: Ground Truth).

	t = 2	t = 85	t = 200
**SiamFC + S+H** **(Tai et al.)** [23]	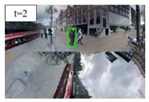	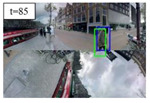	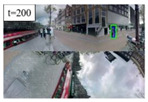
**SiamFC + S+P** **(Proposed Scheme)**	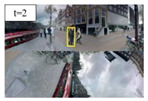	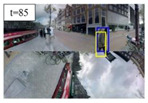	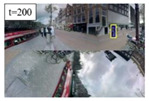

**Table 8:** Tracking speed of all considered trackers in Section 4. Before tracking, videos #1 and #5 are downsampled from 3432 × 2288 to 438 × 292, and videos #2, #3, #4, #6, and #8 are downsampled from 2800 × 1920 to 444 × 296, respectively. Video #7 with spatial resolution 1764 × 1176 is not downsampled. (S: Face stitching, H: Heuristics-based timing detector, P: Machine learning-based timing detector).

Tracker	Videos #1–#6, #8	Videos #7
SiamFC [12] +S	62.35 fps	23.42 fps
Tai et al. [23] (SiamFC + S + H)	60 fps	20 fps
Proposed Tracker (SiamFC + S + P)	52.9 fps	11.2 fps
SiamFC [12] +S +Timing Detector of Template Update of LSSiam [15]	55.95 fps	10.53 fps
SA-Siam [16] +S	50.16 fps	11.7 fps
SA-Siam [16] +S + P	46.04 fps	9.78 fps

## Data Availability

Not applicable.
